# Associations of chronic conditions, APOE4 allele, stress factors, and health behaviors with self-rated health

**DOI:** 10.1186/s12877-015-0132-y

**Published:** 2015-10-26

**Authors:** Wen Hu, Jiehua Lu

**Affiliations:** Department of Social Work, Zhou Enlai School of Government, Nankai University, Tianjin, 300071 China; Department of Sociology, the University of North Carolina, Chapel Hill, NC USA; Department of Sociology, Peking University, Beijing, 100871 China

**Keywords:** Self-rated health, Chronic diseases, APOE4, Stress factors, Health behaviors

## Abstract

**Background:**

Self-rated health (SRH) has been widely used to measure the overall health status of older adults. Research has shown that SRH is determined by a large array of factors, such as chronic disease conditions, genetic markers (e.g., Apolipoprotein E, APOE, NM_000041), stress factors, and health behaviors. However, few studies have incorporated these factors simultaneously in the analytic framework of SRH. The aim of this study is to examine the associations of these four sets of factors with SRH.

**Methods:**

Using a dataset from a population-based, random-cluster survey of 1,005 elderly respondents aged 54–91 conducted in Taiwan in 2000, we use logistic regressions to examine associations of chronic health conditions, the APOE4 allele stress factors, and health behaviors with SRH. The four disease conditions include diabetes, heart diseases, gastric ulcers, and chronic obstructive pulmonary disease. Stress factors are measured by traumatic events (having an earthquake-damaged house) and chronic life stress (financial difficulty). Health behaviors include smoking, drinking alcohol, vegetable and fruit intake, daily milk intake, and physical exercise.

**Results:**

Diabetes, heart diseases, gastric ulcers, and chronic obstructive pulmonary disease are found to be associated with 2.63 (95 % CI: 1.75–3.95), 1.72 (95 % CI: 1.15–2.58), 1.94 (95 % CI: 1.35–2.80), and 2.54 (95 % CI: 1.66–3.92) odds ratios of poor SRH. The APOE4 allele is found to be significantly associated with poor SRH with odd ratio of 1.58 (95 % CI: 1.02–2.41). Financial difficulty is associated with increased likelihood of poor SRH, with odds ratios of 1.76 (95 % CI: 1.22–2.54) Doing exercise more than 5 times per week are associated with reduced likelihood of poor SRH by 44 % (odds ratio is 0.56, 95 % CI: 0.39–1.82). The interaction term between gender and gastric ulcer showed that the impact of gastric ulcer on SRH is more pronounced in women than in men, with an odds ratio of 2.63 (95 % CI: 1.24–5.58).

**Conclusions:**

Chronic conditions and the APOE4 allele are significantly associated with increased likelihood of reporting poor health, and the associations appear differently among women and men. To better understand the mechanism of how people self-assess their overall health, chronic conditions and genetic components should be considered together with conventional factors such as life stress and health behaviors.

## Background

Self-rated health (SRH) is one of the most widely used indicators in population health research [1, 2] and is assumed to be a valid measure of the overall global assessment of a person’s current well-being [3]. Traditionally, medical examination data are considered the “gold standard” to measure health or predict disability [4]. However, it has been pointed out that SRH can sometimes be a better predictor of well-being than biological markers and SRH data can be readily collected for large numbers of individuals at minimal cost [5]. The predictive strength of SRH is its multidimensional measurement of healthy aging that incorporates a wide variety of factors [6].

Previous research has found that both chronic life stress and stress from a traumatic shock are strongly associated with poor SRH [7]. In early studies before the 1980s, the majority of research in this area focused on acute stressful life events – for example, natural disasters, combat, and physical assaults [8–11]. However, as modern life fills with increasing pressures, research has drawn attention to the influence of chronic stressors on health outcomes [12–14]. Measures of chronic life stress generally assess the impact of stressors that last for prolonged but often unspecified periods – for example, low socioeconomic status (SES), or poor working condition or long hours [15, 16].

In addition to stress factors, health behaviors have a significant impact on health and are typically measured by factors such as one’s physical activity, diet, alcohol intake, and use of tobacco products. Specifically, eating fruits and vegetables is positively linked to good health [17], while smoking and heavy use of alcohol have a negative impact on health [18]. Physical exercise promotes health by improving physical and cognitive function [19, 20] and provides a means of socializing in an environment charged with positive emotional content [21].

Chronic diseases (i.e., coronary heart disease, diabetes, and depression) tend to be prevalent among older adults and are evidenced to be closely linked with poor SRH among the elderly [22–24]. People with chronic diseases can experience pain and disability that leads to poor SRH. For example, Research has reported that the presence of chronic diseases is one of core predictors of SRH [25, 26].

SRH also has a genetic basis. Nearly a third of the variability in SRH can be attributed to genetic factors [27]. As noted by Zhang et. al (p1), “Integrating genetic markers into population health research will contribute to a better understanding of the mechanisms through which social and behavioral factors affect population health” [7]. Some studies have examined the association of genetic markers such as the Apolipoprotein E4 (APOE4) allele with health outcomes. For example, research has shown that carriers of the APOE4 allele have an increased risk for earlier age of onset of Alzheimer’s disease and Parkinson’s disease [28, 29], as well as decline in cognitive function and functional status among older adults without dementia [30]. These findings suggest that the APOE4 allele plays an important role in SRH.

However, one shortcoming of the existing literature is that few studies have incorporated chronic diseases, genetic markers, social environmental factors, and health behaviors simultaneously in their analytic framework of SRH. This study attempts to incorporate the APOE4 allele and chronic disease information together with stress factors and health behaviors into research on SRH. We aim to provide a deeper examination of self-rated health among an aging population. The data are from a population-based survey of older adults in Taiwan with a broad range of information including not only self-reports of physical, psychological, and social well-being, but also extensive clinical data based on medical examination and laboratory analyses.

## Methods

### Dataset

The data used in the study were from the Social Environment and Biomarkers of Aging Study (SEBAS) in Taiwan, collected from a representative subsample randomly selected from the 1999 Taiwan Longitudinal Study of Aging (TLSA). The Bureau of Health Promotion of the Department of Health in Taiwan granted approvals for the protection of human subjects for SEBAS. Data were collected through home visits by interviewers. A written consent was obtained from each of all participants with the rare exceptions when a participant who could not read or write. In that case, a consent form was read by the interviewer and signed by a witness.

The 2000 wave of the SEBAS (hereafter the SEBAS 2000) was conducted from July through December 2000 using 27 original primary survey units (PSU) from the TLSA and 10 new townships [31]. All respondents residing in a given PSU were selected for interviews. Data were collected through face-to-face interviews using a structured questionnaire, physical examination, and bio-specimen collection. This human subjects research was approved by the institutional review boards at Princeton University, RAND, Georgetown University, and the Bureau of Health Promotion in Taiwan.

The SEBAS 2000 in-home interview was conducted by a public health nurse who was well-known and highly-respected locally. The questionnaire covered chronic conditions, physical functioning, psychological well-being, cognitive capacity, utilization of health services, and social networks/support. The interviewer then evaluated each respondent’s health. Among the 1,497 respondents, 1,023 received physical exams at a nearby hospital several weeks later (111 were not eligible for health examination and 363 refused to participate). After list-wise deletion, of the 1,023 individuals who completed the survey, 1,005 subjects without any missing data were included in statistical analyses. The age range of participants was 54–91, with 628 persons aged 65 or older and 421 women.

Biospecimens were collected by survey staff during the in-home interview and related the hospital visit. Survey staff collected the 12-hour urine specimen at the participant’s home and accompanied the participant to the hospital on the morning of the scheduled appointment. During the hospital visit, participants were asked about their health history, family disease history, health-related behaviors, and current long-term medications. Blood pressure and anthropometric measurements (i.e., respondent’s height, weight, waist and hip circumference) were performed and a blood specimen was taken to measure biomarkers.

Union Clinical Laboratories (UCL) took responsibility for immediate shipment of the specimens to their headquarters in Taipei, followed standard laboratory protocols for conducting assays, and provided the results to the Bureau of Health Promotion (BHP, in the Department of Health of Taiwan) within two weeks. One genetic marker, the APOE gene, was also obtained by blood specimen using the polymerase chain reaction amplification refractory mutation system (PCR-ARMS) and polymerase chain reaction restriction fragment length polymorphism (PCR-RFLP) analysis. Data quality evaluations conducted during and after the fieldwork by BHP indicate that the SEBAS 2000 rendered reliable data [32].

### Dependent variable

The measure of self-rated health status was based on a simple question: “Regarding your current state of health, do you feel it is excellent, good, average, not so good, or poor?” A binary measure of poor SRH was coded for logistic regression analyses (1 = not so good or poor SRH, 0 = excellent/good/average SRH). Other ordinal categorizations were also tested and the conclusions were very similar.

### Independent variables

The SEBAS 2000 measured fourteen diseases or conditions: high blood pressure, diabetes, heart diseases, stroke, cancer/malignant tumor, bronchitis/emphysema/pneumonia/lung disease/asthma and other lower respiratory tract diseases, arthritis/rheumatism, gastric ulcer/stomach ailment, liver/gallbladder disease, hip fracture, cataract, kidney disease, gout, and spinal/vertebrae spur. All the chronic diseases were self-reported in response to the question “Have you ever had this disease?” Each condition was coded 1 if an individual said yes and 0 otherwise. For this analysis we only included chronic diseases with a prevalence rate of 10 % or higher and with a significant bivariate association with SRH. While hypertension was frequent, it was found to be underreported through evaluation of the SEBAS 2000 with other survey datasets and validation with laboratory results [33]. Eventually, only four diseases, diabetes, heart diseases, gastric ulcer, and chronic obstructive pulmonary disease (COPD) were used to measure physical health in this study.

All respondents who reported having one of these conditions at the time of the interview indicated that a physician delivered the diagnosis (94.7 % for heart diseases, 97.3 % for diabetes, 88.5 % for COPD, and 87.7 % for ulcer). The prevalence rates of these four diseases in SEBAS 2000 were similar to those found in the National Health Interview Survey in Taiwan in 2001 [34]. Moreover, the evaluation of the SEBAS 2000 revealed that, with the exception of hypertension, the accuracy of self-reported chronic disease information in the Taiwan study was similar to that in the United States [33].

The information of the APOE gene, which was obtained from blood specimens analyzed for allele variant, had three alleles: E2, E3 and E4. A binary measure of the APOE4 allele was coded as 1 if the individual carries one or two copies of the E4 allele (carrier), and 0 otherwise (non-carrier).

Stress was measured by traumatic events and chronic life stress. Experiencing housing damage during the 1999 earthquake, which occurred a year before the survey was conducted, was used to measure life stress due to a traumatic event. Environmental stress such as financial difficulty was a major element of psychological stress and was used to measure chronic stress.

The 1999 earthquake was the greatest disaster in late 21st century in Taiwan. It occurred in Jiji, Nantou County, Taiwan on Sept. 21, 1999. Some 2,415 people were killed and 11,305 were injured. The “Quake of the Century” had a profound effect on the whole island, and even on some mainland provinces. The Richter magnitude scale of the 1999 earthquake ranged from 4 in the south (Kaohsiung) to 5 in the north (Taibei) and east (Hualian) and 7 in the west (Yunlin and Jiayiin). Survey participants were asked “Was there any damage or loss to the house in which you usually lived prior to the earthquake?” Response categories were yes (1) and no (0).

Financial condition was measured by the question “Do you (and your spouse) have enough money or any difficulty meeting monthly living expenses or other expenditures?” Possible responses were: “1 = enough money, with some left over; 2 = just enough money, no difficulty; 3 = some difficulty; and 4 = much difficulty.” Financial difficulty was coded 1 if the individual selected the third or fourth categories, and 0 otherwise.

Health behaviors included smoking, drinking alcohol, physical exercise, and diet. Four health behaviors were recoded into binary variables (1 = yes, 0 = no) measured by the following questions: “In the past six months, did you smoke?” “In the past six months, did you drink alcohol?” “Do you drink milk every day?” and “Do you eat at least three servings of vegetables and two servings of fruit every day?” For physical exercise, a three-category option of frequency (<=1, 2–5, and 6+) was designed in the questionnaire, and we directly used its categorization without any modification.

To obtain robust results, we controlled for socio-demographic factors in the statistical analyses. Socio-demographic variables included gender, age, marital status, ethnicity, urban or rural residence, education, and occupation. We also included obesity as a measure of physical condition. In accordance with WHO’s criterion of body-mass index (BMI) for Asian populations, we defined obesity as a BMI greater than 23, weight/height (kg/m) [35].

Due to a documented relationship with SRH, disability in instrumental activities of daily livings (IADL) was also included in the analysis [36]. IADL disability involved limitations on buying personal items, managing money/paying bills, riding bus or train by oneself, doing physical work at home, doing light tasks at home, and making phone calls. Respondents were coded as IADL disabled (1) if the individual had at least some difficulty with one or more items, and 0 otherwise.

### Statistical analysis

After examining descriptive statistics (Table 1), we estimated a logistic regression model of the associations of stress factors and health behaviors with odds of poor SRH while controlling for socio-demographic factors (Model I in Table 2). We then incorporated chronic diseases and the APOE4 allele to examine how measures of physical health and genetic information may alter the associations in Model I (Model II, Table 2). Finally, because of significant gender differences in the relative importance of factors associated with SRH [37], we examined all interactions between gender and chronic diseases, the APOE4 allele, stress factors, and health behaviors. However, only one interaction (between gender and ulcer) was significant. We thus analyzed a model that included this interaction in addition to all factors in Model II (Model III, Table 2).

**Table 1:** Proportion of poor self-reported health by study factors

Variable	Code	N	Poor SRH%	*p*
Overall	--	1,005	26.97	
Number of chronic diseases	0	491	17.11	***
	1	379	28.50	
	2	112	57.14	
	3+	23	60.87	
Diabetes	No	848	23.47	***
	Yes	157	45.86	
Heart diseases	No	840	24.17	***
	Yes	164	40.85	
Gastric ulcer	No	780	24.23	***
	Yes	225	36.44	
COPD	No	875	24.00	***
	Yes	130	46.92	
APOE4 allele carriers	No	862	25.99	
	Yes	143	32.87	
Financial difficulty	No	753	23.64	***
	Yes	249	37.35	
Having an earthquake-damaged house	No	882	25.74	*
	Yes	123	35.77	
Smokers	No	783	28.99	*
	Yes	221	19.91	
Daily alcohol use	No	937	27.43	
	Yes	68	20.59	
Daily milk intake	No	608	27.47	
	Yes	395	26.33	
Vegetable and fruit diet	No	474	32.28	***
	Yes	529	22.31	
# of times doing exercise/week	<=1	392	35.04	***
	2–5	198	25.25	
	6+	415	20.00	
Controls				
Gender	Men	584	23.29	**
	Women	421	32.07	
Age	<70	433	29.56	
	> = 70	572	25.00	
Marital status	Others	291	31.96	*
	Currently	714	24.93	
Ethnicity	Fujian	712	27.39	
	Hakka	122	24.59	
	Mainlander	171	26.90	
Rural residence	No	697	28.41	
	Yes	308	23.70	
Education	None	330	36.97	***
	Primary	407	25.06	
	Secondary	197	17.26	
	College+	71	18.31	
Occupation	Professionals	58	15.52	
	Clericals	215	22.79	
	Blue-collar workers	348	28.45	
	Farmers	384	29.69	
IADL disabled	No	701	18.69	***
	Yes	304	46.05	
BMI scores	<=23	939	26.30	
	>23	66	36.36	

(1) COPD = chronic obstructive pulmonary disease. (2) P-value reports significance of chi-square test of difference in proportion between reference categories. (3) *p < 0.05; **p < 0.01; ***p < 0.001

**Table 2:** Odds ratios of poor SRH for chronic diseases, APOE4 allele, stress factors and health behaviors

	Model I			Model II			Model III		
	OR	95 % CI		OR	95 % CI		OR	95 % CI	
Chronic Diseases									
Diabetes (no)				2.63	1.75–3.95	***	2.63	1.75–3.95	***
Heart diseases (no)				1.71	1.14–2.56	**	1.72	1.15–2.58	**
Gastric ulcer (no)				1.97	1.37–2.84	***	1.94	1.35–2.80	***
COPD (no)				2.57	1.67–3.95	***	2.54	1.66–3.92	***
APOE4allele (no)				1.55	1.01–2.39	*	1.58	1.02–2.41	*
Stress									
Financial difficulty (no)	1.69	1.19–2.40	**	1.77	1.23–2.56	**	1.76	1.22–2.54	**
Having an earthquake-damaged house (no)	1.56	1.01–2.43	*	1.50	0.94–2.39		1.50	0.94–2.39	
Behaviors									
Smoking (no)	0.65	0.41–1.01		0.65	0.41–1.03		0.65	0.41–1.03	
Daily alcohol use (no)	1.00	0.51–1.97		1.05	0.53–2.09		1.01	0.51–2.01	
Vegetable and fruit diet (no)	0.78	0.57–1.07		0.75	0.54–1.05		0.76	0.54–1.06	
Daily milk intake (no)	1.12	0.80–1.57		1.13	0.80–1.61		1.15	0.81–1.63	
Doing exercise 2–5 times/week (<=1 time)	0.78	0.51–1.18		0.70	0.46–1.08		0.72	0.47–1.11	
Doing exercise 5+ times/week (<1 time)	0.56	0.39–0.79	**	0.55	0.38–0.80	**	0.56	0.39–0.82	**
Controls									
Women (men)	0.95	0.64–1.41		0.93	0.62–1.41		0.95	0.63–1.43	
Ages > =70 (<70)	1.06	0.74–1.51		1.11	0.76–1.62		1.10	0.76–1.60	
Currently married (no)	0.94	0.66–1.33		0.95	0.66–1.39		0.97	0.67–1.40	
Ethnicity Hakka (Fujian)	1.09	0.67–1.78		1.21	0.72–2.01		1.22	0.73–2.04	
Ethnicity Mainlander (Fujian)	1.40	0.87–2.24		1.23	0.75–2.03		1.27	0.77–2.08	
Rural (urban)	0.95	0.67–1.36		0.96	0.67–1.40		0.96	0.67–1.40	
Primary schooling (none)	0.93	0.64–1.36		0.92	0.62–1.37		0.92	0.62–1.37	
Secondary schooling (none)	0.59	0.34–1.03		0.58	0.33–1.05		0.58	0.33–1.04	
College degrees or higher (none)	0.87	0.38–2.01		0.87	0.36–2.06		0.86	0.36–2.05	
Clerical (white collar workers)	1.58	0.67–3.72		1.60	0.65–3.90		1.66	0.68–4.05	
Blue-collar workers (white collar workers)	1.61	0.67–3.89		1.59	0.63–4.00		1.62	0.64–4.09	
Farmers (white collar workers)	1.25	0.51–3.06		1.30	0.51–3.32		1.35	0.53–3.46	
IADL disabled (no)	3.13	2.20–4.47	***	2.65	1.83–3.84	***	2.67	1.85–3.87	***
BMI >23 (<=23)	1.27	0.72–2.52		1.47	0.81–2.66		1.45	0.80–2.61	
Interaction Effect									
Women*Gastric ulcer							2.63	1.24–5.58	*
- Log likelihood	521.2***			488.0***			481.1***		

(1) Odds ratios and 95 % confidence intervals are obtained from binary logistic regressions. The category in the parentheses is the reference group of each variable. n2) *p < 0.05; **p < 0.01; ***p < 0.001

## Results

### Univariate analysis

Table 1 provided the distribution of poor SRH by independent variables: chronic diseases, APOE4 allele status, stress factors, and health behaviors, as well as controls. More than 80 % respondents had at least one chronic disease. Overall, 26.97 % of individuals reported poor SRH, and poor SRH was prevalent in individuals who reported diabetes (45.86 %, *p* < 0.001), heart diseases (40.58 %, *p* < 0.001), ulcer (36.44 %, *p* < 0.001) and COPD (46.92 %, *p* < 0.001). Chi square tests showed that individuals who experienced financial difficulty (37.35 %, *p* < 0.001) and earthquake losses (35.77 %, *p* = 0.019) were more likely to report poor SRH than those without such experiences. Individuals who exercised less than once a week (35.04 %, p < 0.001) or had poor dietary habits (32.28 %, *p* < 0.001) appeared to have a higher rate of poor SRH compared to those with more regular physical activity. Interestingly, cigarette smokers were less likely to report poor SRH (28.99 %, *p* = 0.012) than non-smokers, even though cigarette smoking has been linked to poor health. Next, these bivariate associations with poor SRH need to be tested simultaneously in one model while controlling for potential confounding factors in multiple regression analyses.

### Multiple logistic regression analysis

We first fitted a logistic regression for odds of poor SRH including stress factors and health behaviors while controlling for sociodemographics (Model I in Table 2). The two stress factors, having financial difficulty (OR = 1.69, 95 % CI: 1.19–2.40) and having an earthquake-damaged house (OR = 1.56, 95 % CI: 1.01–2.43), were significantly associated with increased odds of poor SRH. The health behavior of engaging in physical activity 6 times per week or more was significantly associated with decreased odds of poor SRH compared to exercising once a week or less than (OR = 0.56, 95 % CI: 0.39–0.79).

When chronic diseases and the APOE4 allele were added to the regression analysis (Model II in Table 2), financial difficulty and frequent exercise remained significantly associated with SRH, indicating that these associations are independent of chronic disease conditions and APOE4 allele. With regard to association between chronic diseases and SRH, we observed strong associations of all four major chronic diseases with increased odds of poor SRH: diabetes (OR = 2.63, 95 % CI: 1.75–3.95), heart diseases (OR = 1.71, 95 % CI: 1.14–2.56), ulcer (OR = 1.97, 95 % CI: 1.37–2.84) and COPD (OR = 2.57, 95 % CI: 1.67–3.95). The association between APOE4 allele and SRH was also significant, with 55 % higher odds of poor SRH (95 % CI: 1.01–2.39) for carriers compared to non-carriers.

Model III in Table 2 provided results from multiple logistic regressions including an interaction between gender and ulcer. The results showed an odds ratio of 2.63 (95 % CI: 1.24–5.58) for the interaction, suggesting that impact of ulcer on SRH is more pronounced in women than men.

## Discussion

This study provides a deeper examination of self-rated health (SRH) among an aging population by applying a model of genetic, physical, stress, and behavioral factors to a population-based survey of older adults in Taiwan with a broad range of self-reported and clinical data. We find that diabetes, heart diseases, gastric ulcers, and chronic obstructive pulmonary disease are associated with poor SRH as reported in the literature [38–40]. Chronic disease could affect health outcomes in several ways. Chronic disease as a measure of physical health may directly affect individuals’ assessment of overall health [41]. Moreover, chronic disease may result in physiological impairment which limits physical or emotional abilities [42], indirectly increasing the likelihood of poor SRH.

One interesting finding is that the association between gastric ulcer and SRH is stronger in women than in men. This finding may be due to gender-based differences in attitudes toward this chronic health condition [43]. Literature has shown that men seem to worry less about long-term complications compared to women [44]. It is possible that women have a stronger sense to worry about gastric ulcer, which may explain why this disease is associated with poor SRH among women. Such sense could results in gender difference in medical care seeking behaviors [45]. Consequently, women will give more weight to the presence of the disease when they assess their overall health. Nevertheless, more studies are need to verify this finding from different populations to shed the lights to the underlying mechanism.

We further find that the APOE4 allele is significantly associated with increased likelihood of reporting poor health. The APOE4 allele is associated with an increased risk of reporting poor SRH in many ways related to cognitive and functional status. Previous research suggests that APOE might play a neurotrophic function in the central nervous system and that altered functioning of this molecule could result in neurodegeneration [46]. Recent studies have found that the APOE4 allele is significantly and uniquely related to lower cognitive scores, significantly increases odds of cognitive decline, and exacerbates the process of cognitive impairment [28, 29]. Moreover, the presence of the APOE4 allele is also associated with functional deficit, apart from the effects of neuropsychological performance. Therefore, the APOE4 allele is associated with poor SRH directly through cognitive decline and indirectly through functional status.

Stress factors are generally thought to influence health outcomes through negative emotional responses [47]. Our study indicates that stress from traumatic shock (earthquake in 1999) is significantly associated with increased odds of poor SRH among older adults. However, this relationship is explained by the inclusion of chronic diseases. Studies have shown that people who are exposed to traumatic events are at increased risk for somatic symptoms and physical illnesses, as well as for major depression, panic disorder, and generalized anxiety disorder, which lowers reserve capacity of dealing with life stress and shocks from the environment, and eventually suffer from some diseases [47]. Consistent with previous research [7], chronic life stress like financial difficulty is associated with SRH. Social contextual factors, especially socioeconomic status, are sufficiently stable over time for SRH [48]. Not only do environmental conditions operate directly in the causation of stress reactions, but the environmental setting and related socioeconomic status also provide and withhold the resources necessary to draw upon in coping with stress [49].

Negative health behaviors, such as lack of exercise, have been considered major factors in determining health outcomes [17, 19]. Physical activity is related to better functioning and overall health status compared with inactivity. Both cross-sectional and longitudinal studies have reported that physical activity delays the deterioration in health status or is related to a better health status compared with unhealthy behaviors [50, 51]. Some studies argue that people do take their health behaviors into consideration when they rate their overall health condition [52].

In interpreting our results, following limitations should take into account. One is that the data on chronic diseases is self-reported based on a person's own understanding of his or her health, which may not be in accordance with the appraisal of medical experts. Although the two evaluations can certainly be combined, major tension often exists between the two perspectives [53]. Another limitation of the study is the lack of neuropathologic conformation of relationships between the APOE4 allele and SRH and between the four chronic diseases and SRH. Although population-based study has sufficient capability to adjust for risk factors, such as genetic, physical, stress, and behavioral factors, general associations need to be followed up with smaller-scale basic science research. Thus, epidemiological and molecular studies are required to define the precise pathway by which the APOE4 allele and chronic disease confers risk of poor SRH and to determine how and why the relationships differ between women and men. Third, the sample size is not large, which prohibited us from examining SRH and its associated factors by subpopulation. Moreover, our analyses have a cross-sectional nature, which are less robust than those based on longitudinal data in studying SRH. Research using longitudinal datasets is thus preferable.

## Conclusions

Chronic conditions and the APOE4 allele are associated with significantly increased likelihood of reporting poor health, and the associations appear differently among women and men. To better understand the mechanism on how the respondents self-assess their overall health, models of SRH need to consider chronic conditions and genetic components together with conventional factors such as life stresses or behaviors. Examining the relative associations of those factors with health and well-being could help to optimize and target resources and activities. According to our study, intervention programs should focus principally on older people who suffer from chronic diseases. Further research is also needed to examine older adults’ needs stemming from poor SRH in more depth, with a focus on discrepancies between different groups based on social, genetic, or chronic disease information.

## Abbreviations

SRH: Self-rated health
APOE: Apo lipoprotein E
SES: Socioeconomic status
TLSA: Taiwan Longitudinal Study of Aging
SEBAS: Social Environment and Biomarkers of Aging Study
PSU: Primary survey units
UCL: Union Clinical Laboratories
BHP: Bureau of Health Promotion
PCR-ARMS: Polymerase chain reaction amplification refractory mutation system
PCR-RFLP: Polymerase chain reaction restriction fragment length polymorphism
COPD: Chronic obstructive pulmonary
BMI: Body-mass index
IADL: Instrumental activities of daily livings

## References

[1] Garrity TF, Somes GW, Marx MB (1978). Factors influencing self-assessment of health. Soc Sci Med.

[2] Maddox GL (1962). Some correlates of differences in self-assessment of health status among the elderly. J Gerontol.

[3] Adams J, White M (2006). Is the disease risk associated with good self-reported health constant across the socioeconomic spectrum?. Public Health.

[4] Ferraro KF, Su Y (2000). Physician-evaluated and self-reported morbidity predicting disability. Am J Public Health.

[5] Rahman MO, Barsky AJ (2003). Self-reported health among older Bangladeshis: how good a health indicator is it?. The Gerontologist.

[6] Østbye T, Krause KM, Norton MC, Tschanz J, Sanders L, Hayden K, Pieper C, Welsh-Bohmer KA (2006). Ten dimensions of health and their relationships with overall self-reported health and survival in a predominately religiously active elderly population: the cache county memory study. J Am Geriatr Soc.

[7] Zhang F, Lewis M, Yang G, Iriondo-Perez J, Zeng Y, Liu J (2008). Apolipoprotein E polymorphism, life stress, and self-reported health among older adults. J Epidemiol Community Health.

[8] Phifer James F, Krzysztof ZK, Fran HN (1988). The impact of natural disaster on the health of older adults: A multiwave prospective study. J Health Soc Behav.

[9] Hyer L, Summers M, Boyd S, Litaker M, Boudewyns P (1996). Assessment of older combat veterans with the Clinician Administered PTSD Scale. J Trauma Stress.

[10] Golding JM (1994). Sexual assault history and physical health in randomly selected Los Angeles women. Health Psychol.

[11] Dohrenwend BP, Dohrenwend BS (1969). Social status and psychological disorder: A causal inquiry.

[12] Dohrenwend BP, Dohrenwend BS (1974). Social and cultural influences on psychopathology. Annu Rev Psychol.

[13] Finch BK, Kolody B, Vega WA (2000). Perceived discrimination and depression among Mexican-origin adults in California. J Health Soc Behav.

[14] Weitz R (2013). The sociology of health, illness, and health care: A critical Approach.

[15] Turner RJ, Wheaton B, Lloyd DA. The epidemiology of social stress. Am Sociol Rev. 1995;60(2):104-125.

[16] Reijneveld SA (2002). Neighbourhood socioeconomic context and self-reported health and smoking: a secondary analysis of data on seven cities. J Epidemiol Community Health.

[17] Cockerham WC (2006). Health lifestyle theory in an Asian context. Health Sociol Rev.

[18] Rose W. The Sociology of Health, Illness, and Health Care: A Critical Approach (Fifth Edition). Boston:Wadsworth Cengage Learning;2010.

[19] Meisner BA, Dogra S, Logan AJ, Baker J, Weir PL (2010). Do or decline? Comparing the effects of physical inactivity on biopsychosocial components of successful aging. J Health Psychol.

[20] Leung GT, Fung AW, Tam CW, Lui VW, Chiu HF, Chan WM, Lam LC (2010). Examining the association between participation in late-life leisure activities and cognitive function in community-dwelling elderly Chinese in Hong Kong. Int Psychogeriatr.

[21] Brajša-Žganec A, Merkaš M, Šverko I (2011). Quality of life and leisure activities: How do leisure activities contribute to subjective well-being?. Soc Indic Res.

[22] Ramkumar A (2009). Self-rated health, associated factors and diseases: a community-based cross-sectional study of Singaporean adults aged 40 years and above. Ann Acad Med Sing.

[23] Molarius A, Janson S (2002). Self-rated health, chronic diseases, and symptoms among middle-aged and elderly men and women. J Clin Epidemiol.

[24] Teh JKL, Nai Peng T, Sor Tho N (2014). Ethnic and gender differentials in Non-communicable diseases and self-rated health in Malaysia. Plos One.

[25] Verropoulou G (2009). Key elements composing self-rated health in older adults: a comparative study of 11 European countries [J]. Eur J Ageing.

[26] Jie JW, Rochtchina E, Lee AJ, Chia EM, Smith W, Cumming RG, Mitchell P (2007). Ten-year incidence and progression of age-related maculopathy: the blue Mountains Eye Study. Ophthalmology.

[27] Romeis JC, Scherrer JF, Xian H, Eisen SA, Bucholz K, Heath AC, Goldberg J, Lyons MJ, Henderson WG, True WR (2000). Heritability of self-reported health. Health Serv Res.

[28] Fillenbaum GG, Landerman LR, Blazer DG, Saunders AM, Harris TB, Launer LJ (2001). The relationship of APOE genotype to cognitive functioning in older African‐American and Caucasian community residents. J Am Geriatr Soc.

[29] O’Hara F, Yesavage JA, Kraemer HC, Meuricio M, Friedman LF, Murphy GM (1998). The APOE epsilon 4 allele is associated with decline on delayed recall performance in community-dwelling older adults. J Am Geriatr Soc.

[30] Albert SM, Gurland B, Maestre G, Jacobs DM, Stern Y, Mayeux R (1995). APOE genotype influences functional status among elderly without dementia. Am J Med Genet.

[31] Chang MC, Hermalin A. Comparative Study of the Elderly in Four Asian Countries. Ann Arbor, MI: Population Studies Center, University of Michigan; 1989. The 1989 survey of health and living status of the elderly in Taiwan: questionnaire and survey design. Research report 1.

[32] Weinstein M, Goldman N (2003). Social Environment and Biomarkers of Aging Study (SEBAS) in Taiwan, 2000 [ICPSR Study No 3792].

[33] Goldman N, Lin IF, Weinstein M, Lin YH (2003). Evaluating the quality of self-reports of hypertension and diabetes. J Clin Epidemiol.

[34] Health Promotion Administration, Ministry of Health and Welfare, April 28th 2015. https://olap.hpa.gov.tw/Search.aspx?menu=100000000006&KeyWord=%u6162%u6027%u75c5.

[35] Choo V (2002). WHO reassesses appropriate body-mass index for Asian populations. Lancet.

[36] Gama EV, Damian JE, de Molino JP, Lopez MR, Pérez ML, Iglesias FG (2000). Association of individual activities of daily living with self-rated health in older people. Age Ageing.

[37] Lim WY, Ma S, Heng D, Bhalla V, Chew SK (2007). Gender, ethnicity, health behavior & self-rated health in Singapore. BMC Public Health.

[38] Taloyan M, Wajngot A, Johansson SE, Tovi J, Sundquist J (2010). Poor self-rated health in adult patients with type 2 diabetes in the town of Sodertalje: A cross-sectional study [J]. Scand J Prim Health Care.

[39] Ernstsen L, Nilsen SM, Espnes GA, Krokstad S (2011). The predictive ability of self-rated health on ischaemic heart disease and all-cause mortality in elderly women and men: the Nord-Trondelag Health Study (HUNT).[J]. Age & Ageing.

[40] Farkas J, Kosnik M, Zaletel-Kragelj L, Flezar M, Suskovic S, Lainscak M (2009). Distribution of self-rated health and association with clinical parameters in patients with chronic obstructive pulmonary disease.[J]. Wien Klin Wochenschr.

[41] Wang HP (2003). Disease, functional status, and self-rated health in Taiwan elderly: 1989–1996. Taiwanese J Soc Welfare.

[42] Griffith L, Raina P, Wu H, Zhu B, Stathokostas L (2010). Population attributable risk for functional disability associated with chronic conditions in Canadian older adults. Age Ageing.

[43] Davidson KW, Trudeau KJ, Van RE, Stewart M, Kirkland S (2006). Perspective: gender as a health determinant and implications for health education. Health Educ Behav.

[44] Gåfvels C, Lithner F, Börjeson B (1993). Living with diabetes: relationship to gender, duration and complications. A survey in northern Sweden. Diabet Med.

[45] Heitkemper MM, Jarrett ME (2008). Update on irritable bowel syndrome and gender differences[J]. Nutr Clin Pract.

[46] Masliah E, Samuel W, Veinbergs I, Mallory M, Mante M, Saitoh T (1997). Neurodegeneration and cognitive impairment in apoE-deficient mice is ameliorated by infusion of recombinant apoE. Brain Res.

[47] Yehuda R (2002). Post-traumatic stress disorder. N Engl J Med.

[48] Braveman PA, Cubbin C, Egerter S, Williams DR, Pamuk E (2010). Socioeconomic disparities in health in the United States: what the patterns tell us. Am J Public Health.

[49] Lazarus RS, Cohen JB. Environmental stress. In Altman I, Wohlwill J F. (eds.), Human Behavior and the Environment: Current Theory and Researc. New York:Plenum;1977.

[50] Haveman-Nies A, De Groot LC, Van Staveren WA (2003). Relation of dietary quality, physical activity, and smoking habits to 10-year changes in health status in older Europeans in the SENECA study. Am J Public Health.

[51] Khaw KT (1997). Healthy aging. BMJ.

[52] Manderbacka K, Lundberg O, Martikainen P (1999). Do risk factors and health behaviours contribute to self-ratings of health?. Soc Sci Med.

[53] Sen A (2002). Health: perception versus observation: self-reported morbidity has severe limitations and can be extremely misleading. BMJ.

